# A qualitative exploration of barriers and facilitators to fruit and vegetable consumption among Uruguayan adults using the social ecological model

**DOI:** 10.3389/fpubh.2025.1698481

**Published:** 2025-10-30

**Authors:** Leandro Machín, Alejandra Girona, Silvina Salgado, Cecilia Marrero, Ana Giménez, María Rosa Curutchet, Gastón Ares

**Affiliations:** ^1^Instituto de Fundamentos y Métodos, Facultad de Psicología, Universidad de la República, Montevideo, Uruguay; ^2^Unidad Académica Departamento de Nutrición Básica, Escuela de Nutrición, Universidad de la República, Montevideo, Uruguay; ^3^Universidad Tecnológica del Uruguay, Paysandú, Uruguay; ^4^Centro Universitario Regional Noreste, Universidad de la República, Tacuarembó, Uruguay; ^5^Instituto Polo Tecnológico de Pando, Facultad de Química, Universidad de la República, Pando, Uruguay; ^6^Instituto Nacional de Alimentación, Ministerio de Desarrollo Social, Montevideo, Uruguay

**Keywords:** fruits and vegetables, food choice, consumption, eating behavior, pubic policy

## Abstract

**Introduction:**

Fruit and vegetable consumption remains below public health recommendations worldwide. This study aimed to explore perceived barriers and facilitators to fruit and vegetable consumption among adults living in urban areas outside the capital city of Uruguay, and to identify strategies to promote intake.

**Methods:**

The study relied on a generic qualitative study using focus group discussions. Five focus groups were conducted between October 2024 and April 2025 in three cities in Uruguay (Atlántida, Paysandú, and Tacuarembó), involving 50 adults aged 18 to 77 years, diverse in sociodemographic backgrounds. Transcripts were analyzed using qualitative content analysis, combining inductive identification of emergent categories from the interview transcripts with deductive coding based on the levels of influence of the Social Ecological Model.

**Results:**

Although participants widely recognized the health benefits of fruit and vegetable consumption, most reported inadequate intake and described challenges in sustaining regular consumption. Barriers emerged across multiple levels of influence, including individual (e.g., taste preferences, lack of motivation, limited cooking skills), interpersonal (e.g., household composition and income), and the food environment (e.g., high prices, limited variety). Participants proposed a range of strategies to promote fruit and intake, primarily emphasizing nutrition education and social marketing, but also suggesting interventions to enhance convenience, reduce costs, and improve access.

**Conclusion:**

Findings underscore the need for multilevel interventions that move beyond individual-focused strategies to address broader environmental and structural determinants of dietary behavior. Adopting a food systems-oriented approach to fruit and vegetable promotion may enhance the relevance and effectiveness of public health initiatives.

## 1 Introduction

Fruits and vegetables play a fundamental role in promoting health and are central to sustainable food systems (1). Their regular intake is associated with lower risks of numerous non-communicable diseases, including heart disease, diabetes, respiratory conditions, osteoporosis, several cancers, and reduced overall mortality (2–5). Based on this evidence, consuming at least 400 grams daily, excluding starchy roots such as potatoes, is recommended by the World Health Organization (6).

However, intake levels across the globe remain insufficient. A global assessment involving 266 countries estimated that, as of 2010, the average person consumed 81.3 grams of fruit and 208.8 grams of vegetables per day (7). Insufficient consumption of fruits and vegetables has been identified as a contributing factor to 2.8% of global mortality and 1% of the total burden of disease measured in disability-adjusted life years (DALYs) (8).

Encouraging higher fruit and vegetable consumption remains a public health challenge, shaped by a complex interplay of factors that influence eating behavior (9–11). The Social Ecological Model (SEM) offers a valuable framework for understanding the multilevel determinants of dietary behaviors (12). The SEM highlights how individual behaviors are influenced by interpersonal relationships, community settings, institutional structures, and broader policy environments (13, 14). This model provides a framework for identifying strategic leverage points for interventions that extend beyond nutritional education to encompass the structural and systemic determinants of fruit and vegetable consumption (15, 16).

Several studies have identified a range of barriers to fruit and vegetable consumption, including preferences for other foods, affordability constraints, limited cooking skills, restricted physical access, and lack of time (17–24). However, most of this research has been conducted in high-income countries, particularly in North America, Europe, and Oceania. Evidence from other global regions remains limited, especially regarding the underlying motives that shape food choices (9, 25, 26). Given that dietary behaviors are deeply influenced by cultural, social, and environmental contexts (10, 27), there is a pressing need to expand research in diverse settings to better inform context-sensitive interventions (28).

Although several recent studies in Latin America have examined fruit and vegetable consumption, most have relied on quantitative surveys assessing knowledge, attitudes, and self-reported intake (29–35). Qualitative research exploring the multidimensional factors that shape fruit and vegetable consumption remains scarce, particularly among populations living outside capital cities. By focusing on adults residing in medium-sized urban areas across different regions of Uruguay, this study contributes to addressing these gaps. It provides an in-depth understanding of the contextual and structural determinants of fruit and vegetable consumption and identifies locally grounded strategies to promote intake, thereby enriching the regional evidence base needed to inform more equitable and context-sensitive public health interventions.

In this context, the present study aimed to examine the factors that influence fruit and vegetable consumption among adults in Uruguay, a high-income country in Latin America where intake has remained consistently below national and international recommendations for at least the last 20 years (36–38). Adopting a qualitative approach, this study aimed to generate in-depth insights into the fruit and vegetable consumption practices of adults living in Uruguayan cities outside the capital. The specific objectives were: (i) to identify perceived barriers and facilitators to regular intake of fruits and vegetables, and (ii) to explore participants’ own suggestions for strategies and interventions that could support increased consumption. By amplifying the voices of individuals from diverse regional and socio-demographic backgrounds, the study offers a more nuanced and contextually grounded understanding of the challenges and opportunities for promoting healthier dietary habits in a Latin American setting.

## 2 Methods

A generic qualitative design was chosen to explore participants’ experiences, perceptions, and views related to fruit and vegetable consumption in an open and flexible manner, without being bound to the assumptions of a specific qualitative tradition (39). Focus group discussions were selected as the primary method of data collection because they enable the generation of rich, interactive data through group dialogue, allowing participants to build on each other’s ideas and elaborate on shared experiences (40). This data collection approach is particularly suited for exploring socially embedded practices such as food choices, where collective norms, cultural references, and interpersonal dynamics play a significant role.

### 2.1 Participants

Participants were recruited from three cities located outside Uruguay’s capital: Atlántida, Paysandú, and Tacuarembó, situated in the southern, northwestern, and northeastern regions of the country, respectively. These cities were purposively selected to ensure a broad representation of geographic, socio-economic, and cultural diversity within the national context. In each location, a non-probabilistic, convenience-based sampling strategy was employed, supplemented by snowball sampling techniques. Recruitment was facilitated through collaboration with local institutions, such as community organizations, health centers, and educational facilities, which served as initial points of contact.

Inclusion criteria required participants to be 18 years of age or older and to reside in the corresponding city at the time of the study. The groups were designed to include participants with diverse sociodemographic characteristics, aiming to capture a wide range of experiences related to fruit and vegetable consumption. A total of 50 individuals participated, with ages ranging from 18 to 77 years. Detailed sociodemographic characteristics of the participants are presented in Table 1.

**Table 1:** Characteristics of the participants (*n* = 50).

Characteristic	Number of participants	Percentage of participants (%)
Gender		
Woman	34	68
Man	16	32
Other	0	0
Age		
18–34 years old	19	38
35–35 years old	13	26
46–65 years old	14	28
65 + years old	4	8
Education level		
Incomplete secondary school or less	5	10
Complete secondary school	5	10
Incomplete university	14	28
University degree or higher	26	52
Currently working		
Yes	46	92
No	4	8
Number of people in the household		
1	11	22
2	7	14
3	13	26
4	12	24
5 or more	7	14
Children in the household		
Yes	21	42
No	29	58
Car ownership		
Yes	38	76
No	12	24

The number of focus groups was determined based on the saturation criterion (41), which was monitored throughout data collection. A preliminary analysis of the interview recordings by two members of the research team indicated that no new themes emerged after the first two groups, conducted in Paysandú and Tacuarembó, suggesting that data saturation had been reached. To ensure the inclusion of more diverse perspectives, one additional focus group was carried out in each of these two cities and another in Atlántida. Content analysis was completed after a total of five focus groups, confirming saturation.

### 2.2 Data collection

A total of 5 groups were conducted: 2 in Paysandú, 2 in Tacuarembó and 1 in Atlántida. The focus groups were conducted between October 2024 and April 2025 in spaces specifically prepared to ensure comfort, privacy, and minimal distractions. In Paysandú, the sessions were held at the premises of the Technological University of Uruguay (UTEC), with participants of diverse profiles recruited from within the city. In Tacuarembó, the focus group took place in a facility of the Universidad de la República and included a heterogeneous group comprising university students, administrative and service staff, as well as individuals benefiting from social assistance programs coordinated by the Ministry of Social Development. In Atlántida, participants were drawn from an existing group of older adults who regularly met for social and recreational activities.

Each focus group consisted of between 7 and 12 participants. The discussions lasted approximately 45 min and was facilitated by a male member of the research team trained in psychology and with prior experience in qualitative methods and group moderation. To guide the discussions, a semi-structured interview guide was developed collaboratively by the research team based on previous studies exploring barriers and facilitators to healthy eating (42, 43). The guide included a series of open-ended questions designed to stimulate conversation around key themes related to the purchase and consumption of fruits and vegetables (Table 2). Focus groups were conducted in Spanish, the official language of Uruguay.

**Table 2:** Semi-structured question guide used for moderating the focus group discussions.

Questions
Do you eat fruits and vegetables? Which ones? Why do you choose those particular fruits and vegetables? Why do you think you consume fruits and vegetables? Do you think you eat enough vegetables? Why? And what about fruit? Do you think your intake is sufficient? Why or why not? Are you interested in increasing your consumption of fruits and vegetables? Why? What do you think could help you eat more fruits and vegetables? What actions or changes could support that? And how do you think we could increase fruit and vegetable consumption among the Uruguayan population in general?

All sessions were audio recorded with prior written informed consent from participants. Recordings were then transcribed verbatim in Spanish for analysis with the assistance of the software Whisper.

### 2.3 Data analysis

The focus group transcripts were analyzed using a combined inductive-deductive content analysis approach (44). This strategy allowed for the integration of pre-existing theoretical constructs while also enabling categories to emerge organically from participants’ lived experiences and perspectives. Data analysis followed a systematic and iterative process, conducted by two researchers trained in qualitative methods with disciplinary expertise in psychology and consumer behavior.

In the first stage, a deductive reading of the transcripts was conducted to identify content aligned with the two main themes from the interview guide: (i) barriers and facilitators to fruit and vegetable consumption, and (ii) strategies to increase consumption. In the second stage, the researchers independently engaged in inductive coding to capture additional categories that emerged directly from participants’ narratives within each of the primary themes. Coding was carried out using Taguette, an open-source qualitative analysis tool.

Through iterative comparison and discussion, the two researchers refined the thematic structure and resolved discrepancies by consensus. Subsequently, the categories were mapped onto the Social Ecological Model, which provided a structured lens for examining the multiple, interacting levels of influence on fruit and vegetable consumption.

To ensure rigor and trustworthiness, the full research team, diverse in disciplinary backgrounds, gender, age, and life experiences, participated in multiple rounds of reflexive discussion. These sessions were used to interrogate assumptions, surface potential biases, and ensure that the analysis remained grounded in participants’ voices. The collaborative and reflexive process resulted in only minor adjustments to the initial coding.

The analysis was conducted in Spanish and later translated into English for publication. Illustrative quotes were chosen to exemplify each category and were translated from Spanish to English.

## 3 Results

All participants reported consuming fruits and vegetables, though they varied considerably in terms of frequency and quantity. Despite a shared recognition of the health and nutritional benefits of these foods, most participants felt their intake was inadequate. Many described challenges in sustaining regular consumption, citing both personal and contextual barriers.

“I could eat more, but I just feel lazy.”

“Honestly, I eat fruit out of obligation because I know I should, but I could live without it.”

“If I eat fruit in a week, I'm doing well, but there are weeks when I don't eat any”

“I try [to eat fruits and vegetables], but I’m not even close in terms of portions [referring to the recommendations]”

“I eat very little vegetables and fruit”

The following section presents participants’ accounts of the barriers and facilitators influencing fruit and vegetable consumption, followed by their suggestions for strategies to support and promote increased intake.

### 3.1 Barriers and facilitators to fruit and vegetable consumption

Participants identified a range of barriers and facilitators to fruit and vegetable consumption spanning all levels of the Social Ecological Model, as summarized in Table 3. The following sections present a detailed account of these factors, drawing directly from participants’ narratives.

**Table 3:** Barriers and facilitators to fruit and vegetable consumption identified in the focus groups according to the levels of the social ecological model.

Level	Barriers	Facilitators
Individual	- Liking for fruit and vegetables - Preference for other types of foods (e.g., meat, ultra-processed products) - Lack of habit - Lack of positive experiences with fruit and vegetables during childhood - Time scarcity and lack of convenience - Lack of cooking skills	- Aversions or disliking for specific fruits and vegetables - Ingrained habits and positive experiences with fruit and vegetables during childhood - Changes in preferences during adulthood - Organization and meal planning - Health motivation
Interpersonal	- Living alone - Preferences of other family members - Economic constraints	- Presence of children in the household
Food environment	- Price of fruit and vegetables - Limited access to a variety of fruits and vegetables - Lack of availability at kiosks or convenience stores	- Cross-border shopping
Macro-level	- Role of fruits and vegetables in culture and traditional dishes	

#### 3.1.1 Individual-level factors

Participants consistently emphasized individual-level barriers and facilitators to fruit and vegetable consumption, with personal taste preferences, ingrained habits, time constraints and convenience, cooking skills, and health motivation emerging as dominant categories. While liking fruit and vegetables was identified as a facilitator for consumption, many expressed specific aversions or reluctance to consume specific fruit and vegetables due to their sensory characteristics.

“[I eat fruits and vegetables] because I like them”

“I’ve never liked tomatoes, the texture, the flavor, nothing”

“I’m not really into vegetables”

"I’ve tried to eat it because they say it’s healthy, but I don’t really like it. I’m not a big fan"

“There are veggies I don’t like, and it also depends on how they’re cooked”

Some participants attributed their low consumption of fruits and vegetables to a stronger preference for other types of foods. Meat was commonly cited as a preferred alternative to vegetables, often displacing them from meals. Similarly, a preference for ultra-processed products, such as snacks and sweets, was frequently mentioned as a barrier to fruit consumption.

“I don’t eat much vegetables because I eat a lot of meat”

"For example, what happens to me too is, like she says, that I see an *alfajor* [traditional product composed of two cookies with a sweet spread in-between, typically covered with chocolate] and I see a fruit, and I'm a thousand times more likely to grab the *alfajor* than the fruit”

During the focus group discussions, participants pointed to the influence of childhood upbringing and long-standing family food practices. Some described households where fruit and vegetables were consistently available, while others recalled minimal exposure. According to participants, these early experiences shaped preferences and led to ingrained habits related to fruit and vegetable consumption.

“In my family, fruits and vegetables were always present. Since I was little”

“In my house, vegetables are not really part of our routine”

“I didn’t grow up eating vegetables like (name of participant) did”

"In my house, for example, my mother and my grandmother weren’t the type to insist much about that. My father wasn’t either"

Although early experiences were seen as influential, several participants reported more recent changes in their food preferences, which encouraged greater fruit and vegetable intake: “I used to hate vegetables… it’s only been about two years that I started trying to eat them.”

Time scarcity emerged as one of the most frequently mentioned barriers to fruit and vegetable consumption. Many participants described demanding schedules and daily routines that left little time for food preparation. Vegetables were commonly viewed as inconvenient, largely due to the perceived effort involved in washing, peeling, and chopping, factors that often discouraged their regular use in meals. Regarding fruit consumption, several participants pointed out the lack of convenience for on-the-go snacking, noting that certain fruits require peeling or leave hands sticky, making them less practical during busy workdays.

“Sometimes, for example, when I get home tired, I don’t feel like cooking, and that’s when I skip the vegetables”

“Vegetables take too long to cook. Washing, peeling, chopping. It’s a lot of work”

“In my personal experience, it’s also the time it takes. Like, you have to wash the spinach, for example. That preparation time really limits me”

"In my case, I'm actually out and about all day. So it's more practical for me to buy a *pasta frola* [traditional preparation composed of a sweet dough and sweet spread on top] or a sandwich than to take fruit to work. I'm always on the go during the week."

"That issue—the convenience of eating it—also plays a role”

A few participants identified pre-washed or ready-to-eat salad mixes as helpful in overcoming this barrier: “It’s a matter of time, so sometimes my son has to buy me the [ready-to-eat salad] mix, because it’s more convenient for me. It already comes pre-cut.”

Participants also highlighted the time required to purchase fruits and vegetables, along with their short shelf life, as key barriers to consumption.

“They go bad really quickly. For example, if you buy a big bunch and plan to eat it over several days, they go bad really fast. They spoil very fast"

"The fruit goes bad faster than vegetables, and I eat just a little. So, I’d rather not eat [fruit] at all"

To address these challenges, some participants described adopting strategies such as weekly meal planning and purchasing or storing frozen fruits and vegetables.

“Setting aside some time one day a week to get everything prepared, even just part of it, helps a lot”

"What I do, for example, is to buy a lot of frozen vegetables, not sure if it’s good, but twice a week I boil everything I buy, all kinds, everything I find, and keep it in the fridge."

“I’ve been eating a lot of frozen fruit … because fresh fruit doesn’t last at home, so I buy and freeze it”

Participants also identified limited cooking skills and lack of confidence in food preparation as important barriers to fruit and vegetable consumption. While some had developed new habits through learning and experimentation, others admitted avoiding vegetables due to uncertainty about how to prepare them in appealing ways.

“I try to make something, and it turns out terrible, you know? Then I end up eating a hamburger”

"Some people don’t like vegetables or don’t eat them because they don’t season them properly"

“I started eating more vegetables once I learned how to use more variety”

Health issues, both personal and within the family, played a motivating role in fruit and vegetable consumption for many participants. In addition to physical health, a few participants also linked fruit and vegetable intake to mental well-being, describing improvements in mood, energy, and emotional stability. Notably, health-related motivations were often reactive rather than proactive, typically triggered by a medical diagnosis or recommendation. Several participants described adopting healthier eating habits in response to chronic conditions such as diabetes, cancer, or the need to manage their weight.

“It’s good to eat vegetables because of the vitamins they provide”

“I struggled with acne for a long time … increasing my intake of fruits and vegetables made a noticeable difference”

"It even affects psychological and emotional well-being, doesn't it?"

"When I eat fruits and vegetables, I feel lighter and more energetic"

“Lately, my mother started having health problems from not eating fruits and vegetables, along with another issue, so we’ve been trying to gradually introduce vegetables, slowly adding lettuce, tomato, onion, and fruits as well"

“I believe this helps with diabetes, and that’s why I had to change my diet”

“[I eat fruits and vegetables because] I have cancer”

“Well, bananas were one of the key things because they have a lot of potassium. Yeah. Because I used to get leg cramps for a long time”

#### 3.1.2 Interpersonal-level influences

Interpersonal-level factors were also discussed in the focus groups, though less frequently than individual or environmental influences. Family composition emerged as an important element shaping fruit and vegetable consumption. Having children in the household was often described as a facilitator, with participants emphasizing the importance of supporting their growth and development and the desire to instill healthy eating habits from an early age. Conversely, some participants described challenges related to household dynamics, such as having to navigate the food preferences or resistance of other family members. Living alone was also identified as a barrier, primarily due to a lack of motivation to cook and concerns about food waste.

“I eat more fruit because of my daughter. It’s something I try to keep at home for her”

“I have two little girls, and I need them to eat, and the two of us join in to help create a habit”

“It’s hard to eat well when others in the household don’t eat the same way”

“If I live alone and make a salad, I end up throwing away the rest. It’s not worth it”

Economic constraints within the household emerged as a relevant barrier to fruit and vegetable consumption. Some participants described periods of limited income and identified reducing their intake of fruits and vegetables as a coping strategy during those times.

“Sometimes when there’s no income at home, it’s difficult [to eat fruits and vegetables]”

"I went a long time without work. And well, there was nothing at home. You have to make do with what you have"

#### 3.1.3 Food environment-level influences

The price of fruits and vegetables emerged as a central concern across all focus groups. Participants consistently perceived these foods as expensive, especially when compared to cereals and energy-dense, ultra-processed products. Fluctuating prices and significant differences between food outlets—such as supermarkets versus farmers’ markets—fueled frustration and uncertainty around purchasing decisions.

“Those products, fruits and vegetables, have a big impact on the budget. To me, they’re relatively more expensive than a bag of rice. So, if we want to talk about consumption, I think cost is a barrier, and it’s only going to become more significant over time”

“If you compare fruit to a chocolate bar, chocolate is always cheaper. Always. A chocolate bar or any ready-made food is always less expensive”

“For me, the main issue is how much prices vary. Sometimes apples cost 2 pesos, and other times you go to buy a kilo and it’s 200 more”

“At the farmers’ market, tomatoes are 45 pesos per kilo, but in the supermarket they’re 120”

Notably, participants from Paysandú, a city bordering Argentina, mentioned cross-border shopping as a strategy to stretch their food budgets: “I used to afford a full month’s supply with the same budget. Now, I have to go across the border to buy groceries.”

Concerns about affordability were expressed by individuals from diverse socioeconomic backgrounds, suggesting that price-related barriers are not solely driven by material constraints, but also by subjective perceptions of value and cost–benefit. In this regard, a couple of participants acknowledged that ultra-processed products are also expensive but are still purchased, as exemplified in the following quote: “I’m not sure if they [referring to ultra-processed products] are cheaper. I think they are really expensive, but we buy them anyway.”

Limited access to a wide variety of fruits and vegetables was frequently cited as a barrier to consumption. Some participants pointed to seasonal limitations and Uruguay’s climate as contributing factors, while others emphasized that good-quality, diverse, and affordable produce was harder to find in the cities farther from the capital. In addition, participants noted that fruits and vegetables were typically only available at specific outlets, such as produce markets or larger stores, whereas convenience stores and kiosks rarely offered healthy options. This limited availability made it easier to purchase processed snacks than fresh fruit, reinforcing unhealthy eating habits in everyday routines.

“The thing is, we're very limited to seasonal fruits. There are other places where, honestly, what they call seasonal fruits are actually available all year round”

“Here in Tacuarembó, people eat very little fruit—always the same ones: apples, bananas, and oranges”

“We have some variety, but not everything that’s available elsewhere”

"In Paysandú, there aren’t very good large fruit shops or big stores that offer a variety"

"But it’s not easy either, because I walk by, see a kiosk, and buy some junk food and an *alfajor*, but I can’t find a kiosk where I can buy an apple. I have to go to a fruit shop. Just look at the options available to you"

#### 3.1.4 Macro-level factors

Participants also discussed cultural attitudes that influence fruit and vegetable consumption in Uruguay. For many, vegetables were viewed as side dishes rather than main components of meals. Traditional diets and limited familiarity with a wide variety of vegetables shaped how fruit and vegetables were perceived and prepared. This cultural framing often reduced their prominence on the plate, reinforcing habitual consumption of a narrow range of familiar options.

"For me, vegetables and salad were always more of a side dish, not a main meal"

"So there’s also a cultural aspect, because usually when you think of broccoli or cauliflower, it’s just a salad. But there are other ways to prepare them that are much tastier. Definitely."

"I feel like we Uruguayans are more traditional. Potatoes, sweet potatoes, maybe some carrots, lettuce, and tomato — and that’s about it, right? So when it comes to serving eggplant, zucchini, or all those other types of vegetables, I think we eat them much less. Same with broccoli and cauliflower"

### 3.2 Suggestions for promoting fruit and vegetable consumption

Participants proposed a range of strategies to promote greater fruit and vegetable intake, addressing barriers at the multiple levels of the Social Ecological Model. Most suggestions focused on the individual level, aiming to tackle knowledge and motivation barriers. In this regard, the most commonly cited approach operated at the interpersonal and institutional level, emphasizing the implementation of educational initiatives designed to raise awareness of the health benefits of these foods. Many participants highlighted the role of schools and early childhood centers in shaping dietary habits through regular educational activities in early childhood.

“There needs to be more awareness. I’m not sure if that’s exactly how to put it, but there should be more talks about this—reaching people in ways that benefit their health and well-being.”

“At my son’s daycare, for example, a nutritionist used to visit occasionally. One time, she gave a morning talk and invited the parents as well. I thought it was really helpful.”

“Like she said, there were always talks at daycare and school. A nutritionist came once a month to give a talk in the morning and always invited the parents. That would be great. When I was in school, the only one talking about food was the internal staff saying, ‘You can’t eat this or that.’”

“I think we need a more proactive policy in schools, where teachers have discussions about this with the kids”

At the policy or macro-level, one group also highlighted the need for communication campaigns, particularly on social media, to promote fruits and vegetables. Participants noted that children and adolescents are constantly exposed to marketing of unhealthy foods and beverages. They considered it essential to counteract this influence with promotional strategies that normalize and highlight healthy options.

“There really needs to be a presence for all of this [fruits and vegetables] on social media, it’s just not there. In the end, what are kids being offered? Junk. Good habits are totally absent from TikTok”

At the household or interpersonal level, several participants across all focus groups emphasized the importance of improving household meal organization as a key strategy for increasing fruit and vegetable consumption. They noted that planning meals in advance not only helped them to more frequently incorporate vegetables in their meals, but also reduced reliance on convenience foods and last-minute decisions.

“To me, it all comes down to planning”

“If you already have things prepared, it’s easier. But if you come home from work or running errands and there’s nothing ready, you end up eating whatever’s there”

Strategies to make purchasing fruits and vegetables more convenient were also seen as promising avenues. At the level of the food environment, home delivery services and subscription-based models were particularly valued for their potential to simplify access. In addition, participants pointed to the appeal of ready-to-eat or frozen options, which can significantly reduce preparation time and support daily consumption.

“There are people who deliver produce to your home, especially those selling organic vegetables, right?”

“Or you could set up a subscription service that delivers seasonal produce to your house once a week”

“The easiest way to promote this would be selling fruit salads. You buy it, open it, eat it—done”

“I use frozen fruit a lot. Only frozen, actually. Fresh fruit doesn’t last at my place, so I go for frozen”

Some participants mentioned community gardens as a potential strategy to increase access to fresh produce, especially in neighborhoods with limited supply.

“There are no community gardens—that would be amazing. Maybe in a park or some shared space. It would be great. You could go, plant something, and then harvest it later.”

“Because even in a small yard, you can have a garden. You can grow food in different ways, recycling, using bottles, reusing materials. You can grow your lettuce in large containers. I’ve seen it and I’ve helped, because I also work in that area, and they say growing your own food is like printing your own money"

Finally, some participants across all groups highlighted the need for strategies addressing environmental and macro-level barriers. They emphasized the importance of public policies focused on improving employment opportunities, lowering the cost of fruits and vegetables, and increasing their availability in local retail outlets, especially in areas with limited access to a diverse range of fresh produce.

“Lower the prices”

“That there were more job opportunities”

“There should be more access”

“I go to the store, look at the fruit section, there’s so little variety, and I end up leaving with nothing … There really should be more options”

## 4 Discussion

This study qualitatively explored multilevel factors acting as barriers and facilitators to fruit and vegetable consumption among adults living in regions outside the capital of Uruguay, a high-income country in South America. The findings underscore the complex interplay of individual, interpersonal, community, and environmental influences that shape fruit and vegetable consumption, which help explain why it remains persistently low despite widespread awareness of their health benefits. These results are consistent with prior research conducted in high-income countries in North America, Europe and Oceania (18–20, 23), as well as with the broader literature on factors influencing food choice and barriers to healthy eating (9, 11, 21, 24, 43, 45, 46).

By applying the Social Ecological Model, this study was able to capture how barriers and facilitators operate simultaneously across multiple levels of influence, from individual perceptions and skills to structural conditions such as food prices and availability. This multilevel lens provided a more comprehensive understanding of the determinants of fruit and vegetable consumption than would be achieved through analyses focused solely on individual or environmental factors. Overall, the analysis highlights that barriers to fruit and vegetable consumption are both behavioral and structural, reinforcing the need for multilevel strategies that address individual habits alongside broader systemic constraints.

At the individual level, participants’ narratives highlighted a clear disconnection between knowledge and behavior. Although most refer to the nutritional value of fruit and vegetables, consumption was often hindered by dislikes, ingrained habits, lack of time and lack of cooking skills. These findings reflect prior studies indicating that knowledge alone is insufficient to change dietary behavior (11, 47). In this sense, although health-related motivations were highlighted as key facilitators of fruit and vegetable consumption, they often emerged as reactive responses to life-course events, such as a medical diagnosis or a significant health concern, rather than proactive health behaviors, consistent with findings from previous research (48). Parenthood and the onset of health conditions were identified as pivotal moments that spurred participants to increase their intake of fruits and vegetables, echoing trends observed in other studies (48–50). This pattern underscores the importance of encouraging preventive behaviors. Interventions that emphasize the long-term benefits of fruit and vegetable consumption, even in the absence of immediate health concerns, may help foster behavior change.

Personal preferences shaped by childhood exposure and routine consumption were central to participants’ accounts, aligning with literature showing that early experiences critically influence long-term food choices (51–53). In addition, perceived lack of time and difficulty related to preparing or cooking vegetables emerged as another individual-level cited barrier for vegetable consumption. Participants frequently described cooking vegetables as time-consuming and labor-intensive, noting that lack of practical skills, confidence, and culinary inspiration often led them to default to more convenient or familiar options. This finding aligns with previous research indicating that limited cooking skills constitute a significant barrier to healthy eating, particularly regarding the intake of vegetables and whole grains (21, 54–57).

Household characteristics emerged as a key interpersonal-level factor influencing fruit and vegetable consumption. Family composition, such as living alone, having young children, or accommodating the preferences of other household members, shaped eating habits, consistent with existing evidence on the influence of household structure on dietary patterns and health (58–60). In addition to family structure, limited household income was frequently identified as a barrier to purchasing fruits and vegetables. Socio-economic status has been consistently recognized as a major determinant of fruit and vegetable intake in both global and Uruguayan studies (29, 61, 62). In this context, reducing the consumption of fruits, vegetables, and other nutrient-dense foods has been documented as a common coping strategy in response to financial constraints (63–66).

Economic concerns emerged as one of the most powerful and consistent structural barriers across all groups, in agreement with the fact that fruits and vegetables are the largest contributor to the cost of a healthy food basket in Uruguay (67). Participants repeatedly emphasized the high and fluctuating prices of fruits and vegetables relative to other foods, particularly ultra-processed options. These perceptions mirror broader structural inequities in the global food system, where healthier foods are often less accessible and more expensive than their less nutritious counterparts (68, 69).

Another structural barrier for consumption of fruits and vegetables was related to the characteristics of the external food environment. Several participants described their local food environments as limited in both the variety and quality of available fruit and vegetables. These findings are consistent with global data showing that food environments in specific areas often lack diversity, convenience, and affordability (70). In this sense, a recent study conducted in two low-income neighborhoods in Montevideo, the capital city of Uruguay, showed difficulties in accessing variety of good quality foods at affordable prices (71).

Participants proposed a range of strategies to support increased fruit and vegetable consumption, most of which targeted individual-level barriers. Nutrition education and communication campaigns, particularly those beginning in early childhood and implemented through schools and daycare centers, were the most frequently mentioned approaches. This emphasis suggests that participants may underestimate the influence of broader social and environmental determinants on their eating behaviors, even when they acknowledge these factors in their own experiences. This result aligns with a previous study exploring Uruguayan adolescents’ perspectives on promoting healthy eating (72), as well as with broader literature indicating that healthy eating is often framed as a matter of personal responsibility and self-control (73–75).

While individual-focused strategies dominated the discussion, some participants did call for structural interventions, such as lowering the cost of fruits and vegetables, expanding their availability in local retail outlets, and improving employment opportunities. These proposals reflect an important recognition that individual behavior change must be supported by systemic and environmental improvements to foster sustained and equitable access to healthy diets.

### 4.1 Strengths and limitations

A key strength of this study lies in its qualitative design, which enabled an in-depth exploration of the factors influencing fruit and vegetable consumption in a Latin American context—an area where empirical research remains limited (9, 25, 26). By applying the Social Ecological Model as a guiding analytical framework, the study was able to organize participants’ narratives across multiple levels of influence, offering a comprehensive and nuanced understanding of the barriers and facilitators to behavior change. This multilevel perspective is particularly valuable for informing the design of contextually relevant interventions that move beyond individual-level strategies to address broader environmental and structural determinants.

Nevertheless, the study has several limitations. While focus groups are well-suited for eliciting shared experiences and generating rich, contextualized data, they may be subject to group dynamics that inhibit dissenting views or encourage socially desirable responses (40). In addition, the use of voluntary recruitment strategies introduces the potential for self-selection bias, as individuals with a preexisting interest in food-related topics or healthier lifestyles may have been more inclined to participate. Additionally, because the data relied on participants’ self-reported perceptions and behaviors, they may be affected by recall errors or social desirability bias. The relatively small and demographically limited sample constrains the transferability of the findings, as it may not fully reflect the diversity of experiences and perspectives within the broader population. Nonetheless, no substantial differences emerged among participants from the three cities, located in distinct regions of the country, suggesting a degree of consistency among individuals living outside the capital. Future research should build on these findings by including more diverse demographic groups and triangulating qualitative insights with quantitative data to enhance external validity. Moreover, the influence of individual characteristics such as age, gender, socioeconomic status, and household composition on barriers and facilitators to fruit and vegetable consumption warrants further investigation.

## 5 Conclusions and implications

This study highlights multiple leverage points for promoting fruit and vegetable consumption that operate across the different levels of the Social Ecological Model. While participants emphasized the importance of nutrition education initiatives, all demonstrated awareness of the health benefits associated with fruit and vegetable consumption. This suggests that interventions focused solely on information dissemination are unlikely to be sufficient. The findings underscore the value of adopting a multilevel perspective that recognizes the dynamic interplay between individual behaviors, social relationships, environmental conditions, and broader policy and structural determinants. This aligns with growing calls for a comprehensive food systems approach to address dietary behaviors and nutrition-related outcomes (57, 76, 77).

At the individual and inter-personal level, participants frequently identified personal preferences, taste aversions, and a lack of habit or intrinsic motivation as key obstacles to regular fruit and vegetable consumption. These findings point to the potential value of strategies that incorporate tasting opportunities and build culinary confidence through the development of cooking skills and self-efficacy (78–80). Encouraging the use of a variety of preparation techniques could also enhance the appeal and integration of fruits and vegetables into daily meals (78). To address time-related barriers, promoting convenient options such as ready-to-eat, pre-cut, or frozen fruits and vegetables may prove effective. Additionally, emerging evidence suggests that interventions involving food kits or subscription-based models that deliver weekly boxes of fresh produce are positively associated with increased fruit and vegetable intake. Future research could employ participatory and user-centered approaches to co-design such strategies and assess their cultural relevance, acceptability, and feasibility within the Uruguayan context (81, 82).

Results also highlight the importance of addressing the affordability of fruits and vegetables as one of the components of efforts to promote their consumption. The introduction of targeted subsidies for low-income households has been shown to be effective in increasing fruit and vegetable intake, as supported by recent systematic reviews and meta-analyses (16, 83). However, the widespread consensus among participants, regardless of socio-economic background, that fruits and vegetables are expensive suggests that subjective price perceptions may also play a critical role in shaping consumption behavior. In this regard, results from a recent survey conducted among adults in Montevideo (the capital city of Uruguay) indicated that the likelihood of citing price as a reason for perceived insufficient fruit and vegetable consumption did not significantly differ by socio-economic status (29). This underscores the need for further research to better understand how subjective price perceptions are formed in the Uruguayan context. Such insights could inform the design of more nuanced interventions that not only address structural affordability through economic measures but also tackle cognitive and perceptual barriers related to food cost. For example, price communication tools and reference pricing comparisons to help reframe consumer perceptions of the value and affordability of fruits and vegetables.

At the level of the food environment, findings from this study point to the need to enhance access to affordable, good-quality, and diverse fruits and vegetables, particularly in smaller towns. Strengthening local food systems through short supply chains using strategies such as farmers’ markets and supporting community gardens, improve both physical and economic access to fresh produce in underserved areas (84, 85). Participants also emphasized the limited variety available in their local settings and the cultural tendency to rely on a narrow range of traditional fruits and vegetables. Campaigns and interventions aimed at diversifying fruit and vegetable consumption should therefore incorporate culturally adapted messaging and introduce underutilized but accessible produce in appealing ways. From a research perspective, these findings underscore the importance of expanding food environment research beyond the capital city to capture the realities of more geographically and socioeconomically diverse populations. Future studies are necessary to characterize spatial access to healthy foods in the cities where the groups were conducted. Broadening this evidence base is essential to inform equitable food policy and planning that supports fruit and vegetable consumption.

Taken together, these findings offer several concrete directions for public health and food system action in Uruguay. Integrating culinary education and practical food skills into community health programs and school curricula could help translate nutritional awareness into sustained behavior change. At the policy level, implementing targeted subsidies for low-income households could enhance affordability and equity in fruit and vegetable access. Complementary communication campaigns that emphasize convenience, variety, and cultural relevance may help shift perceptions around cost and desirability. Strengthening linkages between smallholder producers and local markets through public procurement programs, regional food hubs, or farmers’ markets could also improve the availability and diversity of produce outside the capital. Finally, aligning these initiatives within a broader national food systems strategy would ensure coherence between public health, agricultural, and social protection policies to promote healthier and more sustainable diets.

## Data Availability

The raw data supporting the conclusions of this article will be made available by the authors, without undue reservation.
